# Which children with chest-indrawing pneumonia can be safely treated at home, and under what conditions is it safe to do so? A systematic review of evidence from low- and middle-income countries

**DOI:** 10.7189/jogh.12.10008

**Published:** 2022-08-31

**Authors:** Chris Wilkes, Hamish Graham, Patrick Walker, Trevor Duke, ARI Review group, Trevor Duke, Hamish Graham, Steve Graham, Amy Gray, Amanda Gwee, Claire von Mollendorf, Kim Mulholland, Fiona Russell, Maeve Hume-Nixon, Saniya Kazi, Priya Kevat, Eleanor Neal, Cattram Nguyen, Alicia Quach, Rita Reyburn, Kathleen Ryan, Patrick Walker, Chris Wilkes, Poh Chua, Yasir Bin Nisar, Jonathon Simon, Wilson Were

**Affiliations:** Centre for International Child Health, Murdoch Children’s Research Institute, University of Melbourne, Royal Children’s Hospital, Parkville, Victoria, Australia

## Abstract

**Background:**

WHO pneumonia guidelines recommend that children (aged 2-59 months) with chest indrawing pneumonia and without any “general danger sign” can be treated with oral amoxicillin without hospital admission. This recommendation was based on trial data from limited contexts whose generalisability is unclear. This review aimed to identify which children with chest-indrawing pneumonia in low- and middle-income countries can be safely treated at home, and under what conditions is it safe to do so.

**Methods:**

We searched MEDLINE, EMBASE, and PubMed for observational and interventional studies of home-based management of children (aged 28 days to four years) with chest-indrawing pneumonia in low- or middle-income countries.

**Results:**

We included 14 studies, including seven randomised trials, from a variety of urban and rural contexts in 11 countries. Two community-based and two hospital-based trials in Pakistan and India found that home treatment of chest-indrawing pneumonia was associated with similar or superior treatment outcomes to hospital admission. Evidence from trials (n = 3) and observational (n = 6) studies in these and other countries confirms the acceptability and feasibility of home management of chest-indrawing pneumonia in low-risk cases, so long as safeguards are in place. Risk assessment includes clinical danger signs, oxygen saturation, and the presence of comorbidities such as undernutrition, anaemia, or HIV. Pulse oximetry is a critical risk-assessment tool that is currently not widely available and can identify severely ill patients with hypoxaemia otherwise possibly missed by clinical assessment alone. Additional safeguards include caregiver understanding and ability to return for review.

**Conclusions:**

Home treatment of chest-indrawing pneumonia can be safe but should only be recommended for children confirmed to be low-risk and in contexts where appropriate care and safety measures are in place.

World Health Organization (WHO) clinical guidelines for children are intended to provide evidence-based guidance to health workers in diverse clinical settings, with a focus on low-resource and smaller health facilities [1-3]. The guidelines for pneumonia were updated in 2013 and changes in severity classification and treatment recommendations for children 2-59 months of age were made [4]. The previous WHO pneumonia classification separated pneumonia into non-severe, severe, and very severe categories [5]. Pneumonia with chest indrawing in children aged 2-59 months was considered at least “severe” with parenteral antibiotics being recommended. The 2013 revision described “pneumonia” and “severe pneumonia” classifications and included children with chest indrawing and no other danger signs (chest-indrawing pneumonia) in the pneumonia group that could be safely managed outside of a hospital [4]. While this change was carried through in revised primary care and hospital guidelines [2,3], the community case management guidelines (iCCM) for community health workers (CHWs) still recommend that CHWs refer children with chest-indrawing pneumonia to a hospital [6].

The 2013 revisions were informed by evidence that oral antibiotics are equivalent to intravenous antibiotics for most children with pneumonia [7-12] and studies demonstrating that many children with “severe” or chest-indrawing pneumonia could be safely treated at home [13-18].

However, the studies which influenced this guideline change were conducted in limited contexts and under controlled trial conditions. The observed pneumonia mortality rates were very low compared to most low- and middle-income country (LMIC) settings, the rates of wheezing and viral aetiology were high, and large numbers of patients were excluded. This raised concern that they may not be representative of the populations for whom WHO guidelines are intended – particularly in higher mortality contexts with higher rates of bacterial pneumonia and in the absence of the level of monitoring usually associated with clinical trials [19]. While these were set up as non-inferiority trials, the dilution of patient populations with children with self-limiting viral lower respiratory tract infections, such as mild-moderate bronchiolitis, risks lowering the power to detect a difference in treatment outcomes for children with bacterial pneumonia, even in very large studies [19].

A subsequent study from Kenya sought to evaluate the appropriateness of this change in guidelines, adding to the evidence showing that chest indrawing in sub-Saharan Africa hospital contexts, along with other factors not currently incorporated in the WHO classification of severity of pneumonia (such as moderate pallor and moderate underweight), were significant risk factors for mortality [20,21].

This review aimed to examine all published studies from LMICs which analysed home treatment of chest indrawing pneumonia, in comparison to referral or admission for inpatient hospital treatment, to establish the degree to which we can be confident in recommending such management, and under what conditions.

## METHODS

### Search strategy

We conducted a systematic search of Medline, Embase, and PubMed (for articles not yet indexed in Medline) for all relevant articles published since January 1, 2000 (search conducted on September 1, 2020). We mapped search terms to medical subject headings where possible, using Boolean operators to combine searches into our final systematic search query. We used synonyms of “pneumonia”, “chest-indrawing”, “home treatment” and “child” to target the search strategy, with oversight from an experienced medical librarian to ensure all relevant papers were identified. We also searched reference lists of all included references for eligible studies. The specific search terms used for our Medline search and further details of the search strategy, information sources, and data collection processes are included in Appendix S1 in the **Online Supplementary Document**.

### Assessment of study eligibility

We included studies published since 2000 evaluating outcomes for children (aged 28 days to 4 years) with WHO-defined pneumonia with chest-indrawing (“chest-indrawing pneumonia”) treated at home (**Table 1**). Two reviewers (CW and PW) independently screened the titles and abstracts of all returned studies. We obtained full texts for studies screened by either reviewer, with the two reviewers independently assessing them for inclusion. We resolved disagreements by discussion and, where appropriate, consulted a third reviewer (HG). No reviewer was blinded to the journal titles, study authors, or affiliated institutions.

**Table 1:** Inclusion and exclusion criteria for studies in this review

Inclusion criteria	Exclusion criteria
Observational or interventional study or meta-analysis involving original data or analysis.	Does not provide original data or analysis (eg, review articles, editorials).
Published in the year 2000 or later.	Does not provide original data or analysis (eg, review articles, editorials).
Published in English.	Conducted in a neonatal unit/neonatal ICU or focuses on infants <28 days of age.
Includes children aged between 28 d and 5 y of age and it is possible to extract data specifically relating to children within these age groups from the data available.	
Includes children whose primary presenting problem was proven or suspected ALRI (which may include both pneumonia and bronchiolitis) with chest-indrawing, and it is possible to extract data specifically relating to these children.	
Examines treatment at home (including community- or facility-based models of care) of children with a primary presentation of chest-indrawing pneumonia-	

ALRI – acute lower respiratory tract infection; LMICs – low- and middle-income countries

We used a standardised data extraction form to extract data relevant to our review. Two reviewers (CW and PW) independently extracted data from each eligible study and entered it into an Excel spreadsheet (Microsoft, Redmond, US). We resolved disagreements by discussion and contacted study authors where appropriate to resolve any uncertainties. We did not attempt a meta-analysis of extracted data as our primary goal was to understand treatment outcomes with respect to population and context. We categorised context, population and outcome data, then qualitatively synthesised results to determine whether children with WHO-defined chest-indrawing pneumonia could be safely managed at home, and in what contexts.

### Assessment of study quality and risk of bias

We assessed the quality and risk of bias of all included studies by using the Effective Public Health Practice Project (EPHPP) Quality Assessment Tool [22,23]. Using this tool, two reviewers, PW and CW, independently rated studies as strong, moderate, or weak with respect to selection bias, study design, confounders, blinding, data collection method, withdrawals and dropouts, and a global rating. Where disagreements occurred, a third reviewer, HG, carried out a final assessment (Table S1 in the **Online Supplementary Document**).

Ethical approval for this study was not required.

## RESULTS

**Figure 1:**
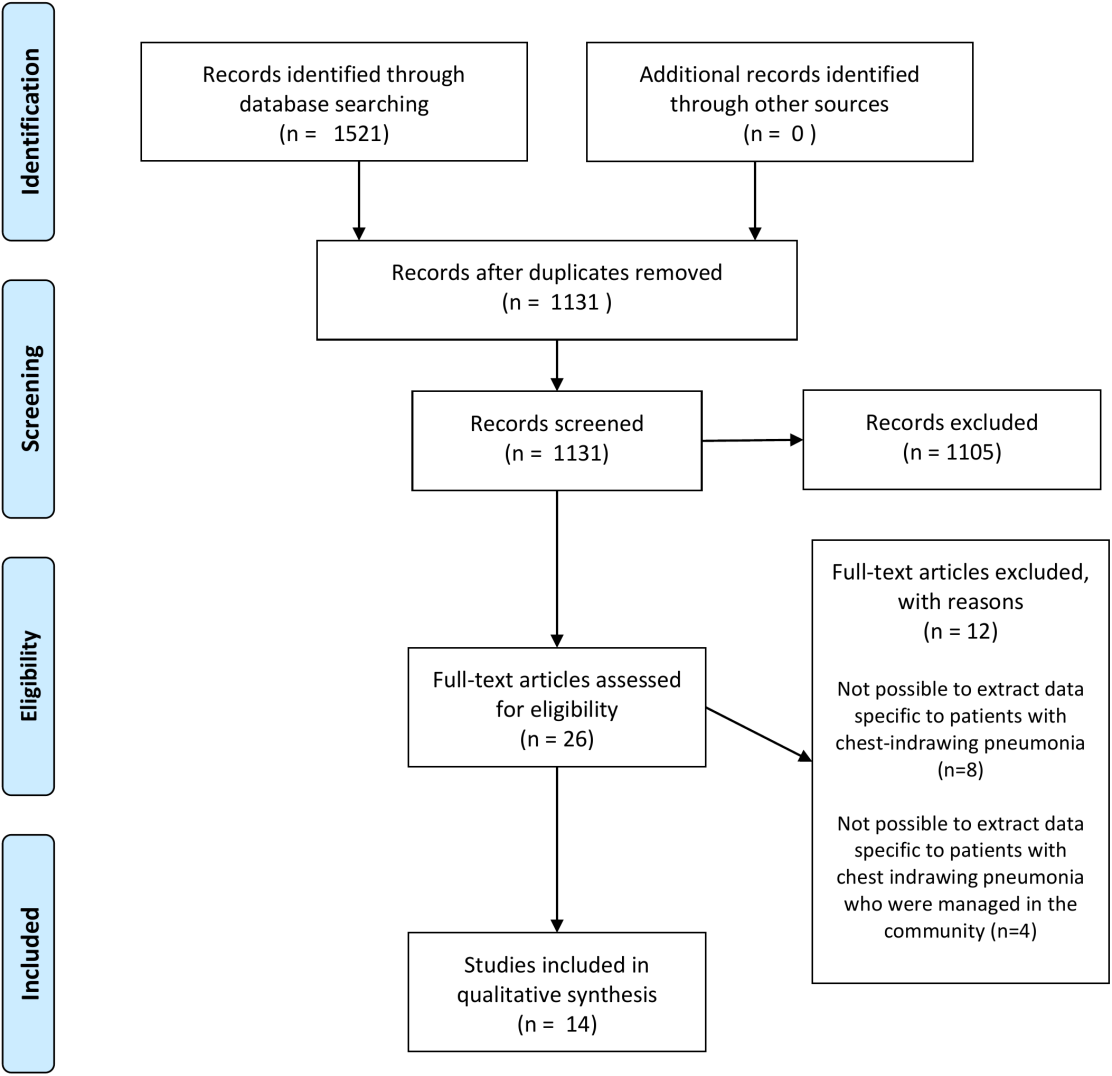
Preferred Reporting Items for Systematic Reviews and Meta-Analyses (PRISMA) flow diagram.

We retrieved 1521 references from database searches, screened 1131 unique articles and identified 26 articles for full-text screening. Of these, 12 were excluded because it was impossible to extract data specific to patients with chest indrawing pneumonia (n = 8) [24-31] or specific to those managed at home (n = 4) [32-35]. We included 14 studies in the qualitative synthesis (**Figure 1**).

### Study characteristics

Characteristics of included papers can be found in **Table 2** and Table S2 in the **Online Supplementary Document**. Of the 14 papers, six were randomised controlled trials (RCTs) [13-15,17,36,43], one was a planned subgroup analysis of a larger RCT [39], six were observational studies [16,18,37,40-42], and one was a case report [38]. Aside from the case report, the number of participants included in each paper ranged from 117 [41] to 13 266 [40]. Half (7/14) of the papers were published after 2015.

**Table 2:** Settings and PICOS criteria of included studies

Paper	No. of patients	Study type	Location of study	Context of study	Inclusion criteria	Key exclusion criteria	Intervention	Comparison	Primary outcome measured
Addo-Yobo 2011 [18]	873	Observational	Bangladesh, Egypt, Ghana, and Vietnam	Facility-based: 3 urban tertiary hospitals, 1 peri-urban secondary hospital, 7 semi-rural primary care centres	Children aged 3 to 59 mo with cough or difficult breathing and lower chest indrawing (non-resolving with salbutamol) without cyanosis or danger signs	Asthma, recurrent wheeze, severe malnutrition, recent hospitalisation, and other disease requiring antibiotics (eg, meningitis, known chronic condition	Outpatient clinic care, oral amoxicillin (80-90mg/kg per day in two divided doses) for 5 d	No comparison group	Cumulative treatment failure by day 6*
Ashraf 2019 [36]	470	RCT	Dhaka, Bangladesh	Facility-based: Urban day clinic and urban referral hospital	Children aged 2-59 mo with cough or difficult breathing with lower chest wall indrawing (non-resolving with salbutamol), **and severe malnutrition** without cyanosis or danger signs	Suspected sepsis, meningitis, convulsions, or other life-threatening illnesses	Day clinic admission (8 am to 5 pm), home overnight, daily IV/IM ceftriaxone (75-100 mg/kg) for 5 d	Inpatient paediatric ward care, daily IV/IM ceftriaxone (75-100 mg/kg) for 5 d	Deaths, discontinuation, referral, and readmission
Ashraf 2008 [37]	251	Observational	Dhaka, Bangladesh	Facility-based: Urban day clinic	Children aged 2-59 mo with cough or difficult breathing with lower chest wall indrawing (non-resolving with salbutamol), **and/or with cyanosis or danger signs** who had been refused admission to local referral inpatient services due to lack of beds	Already taken antibiotics for this illness, associated co-morbidities (eg, TB, CHD, asthma, severe malnutrition, sepsis, convulsion, meningitis) Presented after 14:30	Day clinic admission (8 am to 5 pm), home overnight, daily IV/IM ceftriaxone (75–100 mg/kg) for ≥5 d	No comparison group	Discharged without requiring referral elsewhere
Bari 2011 [17]	3211	Cluster-RCT	Haripur District, Pakistan	Community-based: 511 rural community health workers (LHWs)	Children aged 2-59 mo with cough or difficult breathing and lower chest indrawing (non-resolving with salbutamol) without cyanosis or danger signs	Diarrhoea with severe dehydration, severely malnourished, already on antibiotics	Community case management (LHWs, iCCM) WITHOUT hospital referral, oral amoxicillin (80-90 mg/kg per day or 375 mg twice a day to infants aged 2-11 mo and 625 mg twice a day for those aged 12-59 mo), parental counselling	Community case management (LHWs, iCCM) WITH hospital referral, one dose of oral co-trimoxazole (age 2-11 mo, sulfamethoxazole 200mg plus trimethoprim 40mg; age 12 mo to 5 y, sulfamethoxazole 300 mg plus trimethoprim 60 mg)	Cumulative treatment failure by day 6*
Chowdhury 2008 [16]	1455	Observational (cohort) with pre-post	Matlab Upazilla, Bangladesh	Facility-based: 40 primary care clinics with paramedic health workers	Children aged 0-59 mo with cough or difficult breathing and lower chest indrawing (non-resolving with salbutamol) without danger signs or stridor	Patients with severe pneumonia who also had other severe classifications and those referred for other conditions in need of specialised treatment (eg, trauma or burn)	Outpatient clinic care WITHOUT hospital referral, oral amoxicillin, parental counselling, planned review, referral strengthening	Outpatient clinic care WITH urgent hospital referral, HCW give first dose of antibiotic, ensure proper feeding	Appropriate antibiotic given OR appropriately referred and complied with referral
Hazir 2008 [15]	2100	RCT	Five cities in Pakistan	Facility-based: 7 urban tertiary hospitals	Children aged 3-59 mo with cough or difficult breathing and chest indrawing (non-resolving with salbutamol) without cyanosis or danger signs	Asthma, recurrent wheeze, persistent vomiting, recent hospitalisation, other disease requiring antibiotics	Outpatient clinic care, oral amoxicillin (80–90 mg/kg per day in two doses) for 5 d	Inpatient paediatric ward care, IV ampicillin (100 mg/kg per day in four doses) for 48 h, then oral amoxicillin for another 3 d (80-90 mg/kg per day in two doses)	Cumulative treatment failure by day 6*
Jahan 2018 [38]	1	Case report	Bangladesh	Facility-based: Rural day clinic	N/A – case report of a 27 mo-old child with severe pneumonia with hypoxia		Day clinic admission (8 am to 5 pm), home overnight, IM ceftriaxone 1g daily for 5 d	No comparison group	Discharge in good condition
Keitel 2019 [39]	681	Subgroup analysis of larger RCT	Dar es Salaam, Tanzania	Facility-based: 3 urban secondary hospitals, 6 urban primary health centres	Children aged 2 to 59 mo with cough and 7 d or less of fever, with tachypnoea or lower chest indrawing (non-resolving with salbutamol)	Weight less than 25kg, Signs of severe illness (convulsion or positive meningeal signs, hypoxemia, cyanosis, severe respiratory distress, unable to tolerate oral liquids, severe dehydration, severe anemia, and severe acute malnutrition)	Outpatient clinic care, using e-POCT algorithm to risk-stratify (CRP-informed), oral amoxicillin (80–100 mg/kg/d) for 5 d	Referral to hospital, using ALMANACH algorithm (based on IMCI), all children with chest indrawing given IV ceftriaxone and referred	Cumulative treatment failure by day 7*
McCollum 2016 [40]	13 266	Observational	Lilongwe and Mchinji, Malawi	Facility-based: 18 rural health centres with CHWs	Children aged 2-59 mo with clinically diagnosed pneumonia		Review of effect of measuring oxygen saturation (SpO2) on potential referral rates, compared with Malawian 2000 guidelines which recommend referral due to chest indrawing, and with WHO 2014 guidelines which do not	No comparison group	Numbers of children with Sao_2_ 90%-92% and those with Sao_2_<90%, among all eligible children who would not have been referred if oximetry had been unavailable
Morre 2019 [41]	117	Observational	Port Moresby, Papua New Guinea	Facility-based: urban tertiary hospital	Children aged 1 mo to 12 y with cough and difficult breathing with chest indrawing, without danger signs or hypoxaemia (as measured by pulse oximetry)	Chronic illnesses, including severe malnutrition, tuberculosis, anaemia, HIV, asthma, or chronic lung disease; signs of shock, heart failure (hepatomegaly and heart rate >160) and convulsions; vomiting all feeds or medicine	Outpatient clinic care, stat IM benzylpenicillin (50 000 IU/kg), then home on oral amoxicillin (25 mg/kg 8 hourly) for 5 days	No comparison group	Cumulative treatment failure by day 6*
Onono 2018 [42]	1906	Observational	Homabay county, Kenya	Community-based: rural community health workers	Children aged 2-59 mo with cough or difficult breathing and chest indrawing without cyanosis or danger signs		Community case management (CHW, iCCM), oral amoxicillin (90 mg/kg per day in two divided doses) for five days	No comparison group	1) concordance between CHWs and nurses in identification and classification of lower chest indrawing pneumonia; and (2) cumulative treatment failure by day 4*
Patel 2015 [14]	1118	RCT	6 cities in India	Facility-based: 6 urban referral hospitals	Children aged 3-59 mo with cough or difficult breathing of fewer than 2 weeks duration and lower chest indrawing (unresponsive to nebulised salbutamol) without danger signs, stridor, cyanosis, or Sao_2_<88% in air	Known or clinically recognizable chronic conditions, asthma or recurrent wheeze, respiratory rate (RR)>70, known or suspected HIV, recent hospitalisation, severe malnutrition, convulsions, antibiotic use within 48 h, other diseases requiring antibiotic therapy, persistent vomiting, grunting, severe dehydration, severe pallor, radiological consolidation/effusion/pneumothorax	Outpatient clinic care, first dose of oral amoxycillin (50 mg/kg/d in two divided doses) administered in hospital and subsequent doses were administered by the caregiver at home for seven days	Inpatient hospital care, oral amoxicillin (50 mg/kg/d in two divided doses) in hospital for two days by hospital staff, followed by administration by the caregiver at home for five days	Cumulative treatment failure by day 6 (however, only results for cumulative treatment failure by day 14 presented in the paper)*
Soofi 2012 [13]	4410	Cluster-RCT	Sindh Province, Pakistan	Community-based: rural lady health workers (LHWs)	Children aged 2-59 mo with cough or difficult breathing and chest indrawing (non-resolving with salbutamol) without cyanosis or danger signs	Persistent vomiting, already on treatment for pneumonia, audible wheeze, asthma, severe malnutrition	Community case management (LHW, iCCM), oral amoxicillin (90 mg/kg per day in two doses) by LHWs for 5 d for treatment at home	Referral to hospital, LHW give first dose of oral co-trimoxazole and referred to their nearest hospital for admission and IV antibiotics	Cumulative treatment failure by day 6*
Tesfaye 2020 [43]	1804	Cluster-RCT	Ethiopia, Gedeo Zone	Facility-based: 24 rural primary health centres	Children aged 2-59 mo with cough or difficult breathing for less than 14 d	Diagnosis was for other (non-pneumonia) conditions, such as pulmonary tuberculosis	Outpatient clinic care, IMCI WITH pulse oximeter, oral amoxycillin for chest-indrawing pneumonia without danger signs	Outpatient clinic care, IMCI WITHOUT pulse oximeter, oral amoxycillin for chest-indrawing pneumonia without danger signs	Severe pneumonia diagnosed using the IMCI algorithm in both arms

CHW – community health worker, iCCM – Integrated Community Case Management, IMCI – Integrated Management of Childhood Illness, IM – intramuscular, IV – intravenous, LHW – lady health worker, RCT – randomised controlled trial, mo – months, d – days

*Different studies used varying definitions of treatment failure, but all definitions included clinical deterioration as well as persistence of chest indrawing and/or fever. See main text and Table S3 in the **Online Supplementary Document** for details.

The studies took place in 9 lower-middle income countries and 2 low-income countries. Seven studies were predominately in urban settings [14,15,18,36,37,39,41], and seven were in predominantly rural settings [13,16,17,38,40,42,43]. Three studies involved community-based health workers [13,17,42], eight involved primary care clinics [16,18,39,40,43] or hospitals [14,15,18,39,41], and 3 were of “day clinics” [36-38].

### Patient characteristics

All studies included children 59 months of age or younger, except one [41] which included patients up to 12 years of age. Most inclusion criteria for studies were consistent with pneumonia as per the 2005 or 2014 WHO guidelines [4,5]. Exclusion criteria varied, but children with severe pneumonia (ie, pneumonia with danger signs) were excluded in most studies, as were patients with comorbidities or other conditions for which antibiotics would have been indicated.

Four studies reported vaccination coverage, with generally high coverage ranging from 74% to 97% receiving all age-appropriate vaccines according to local guidelines [14,18,39,43] (Table S2 in the **Online Supplementary Document**).

Many patients were excluded following screening in most of these studies, with numbers of enrolled patients ranging from 5% [16] to 45% [37] of the total number of patients screened. While non-severe or non-chest-indrawing pneumonia was the most common exclusion reason (99.7% of exclusions in one study [16] were due to non-severe pneumonia), other studies had numerous other exclusion reasons, including one in which consent was refused for 61% of screened children [14], one in which 25% of screened patients were excluded for a history of three or more episodes of wheeze [18], and another in which 18% were excluded due to a history of asthma [15] (Table S3 in the **Online Supplementary Document**).

### Objectives of included studies

All studies either compared management of pneumonia with chest indrawing at home vs hospital management, compared management algorithms that mandated hospital management in one arm and allowed home treatment in another, or were observational studies of home care.

### Characteristics of health care resources

Oxygen saturation was documented as having been measured in patients in eight of the 14 studies [14,36-41,43], although in two of these it was only available to patients in one arm of the study [39,43]. All studies had clear protocols for diagnosis, categorisation, management, and referral of pneumonia, though there was variation in how strictly these were adhered to. Most (11/14) studies described follow-up frequency, all requiring review at least twice in the first week, including at least one review by day three (Table S2 in the **Online Supplementary Document**).

### Training and education of caregivers

Ten of the 14 studies described the training procedures for study staff, which ranged from one day [40] to one week [18] and included a variety of techniques, such as lectures, videos, practical sessions, and role play, as well as assessment and ongoing supervision. Six studies described caregiver education, which generally included teaching on how to administer medication and review for danger signs. One study reported the use of a video and formal assessment of understanding (Table S2 in the **Online Supplementary Document**).

### Study outcomes

The primary outcome of eight studies [13-15,17,18,39,41,42] was treatment failure, however varying definitions of treatment failure were used. By all definitions, clinical deterioration, and/or persistence of fever or chest indrawing were considered indicative of treatment failure (Table S4 in **Online Supplementary Document**).

Other studies’ primary outcomes were appropriateness of treatment compared to guidelines [16], successful treatment via a day clinic [36-38], and effect of oximetry on severe pneumonia diagnosis and referrals [40,43].

### Trial evidence on clinical outcomes

Of the seven included randomised or cluster-randomised trials, four [13-15,17] directly compared treatment failure in a community setting with referral or inpatient management of chest-indrawing pneumonia (**Table 3** and **Table 4**). Of these, two studies [13,17] were on “lady health worker” (village-based community health worker) programs in rural Pakistan, and two involved urban tertiary care facilities in Pakistan [15] or India [14]. These four trials found similar or better treatment outcomes for patients in the community management arm relative to the inpatient management or referral arm. The lower risk of treatment failure on intention-to-treat analysis in one trial [14] became insignificant after per-protocol analysis and may have been influenced by numerous patients in the inpatient management arm voluntarily withdrawing from the study and self-discharging from the hospital.

**Table 3:** Outcomes of studies comparing home vs hospital management in which primary outcome was treatment failure in patients with chest-indrawing pneumonia

		Intention to treat (ITT) analysis		Per protocol (PP) analysis			
**Study**	Group*	**Enrolled**	**Cumulative treatment failure^†^ (%)**	**Completed protocol & follow up**	**Cumulative treatment failure† (%)**	**Comparison (95% CI)**	**Mortality by day 6**
Randomised (RCT) and cluster-randomised controlled trials (cRCT)							
Bari 2011 (cRCT) [17]	Intervention	1995	165 (8.3)	1857	165 (9)	PP risk difference = -8 · 91% (-12.38, -5.44)	1 (0.05%)
	Control	1477	241 (16.3)	1354	241/1354 (18)		1 (0.07%)
Hazir 2008 (RCT) [15]	Intervention	1052	77 (7.5)	1025	77 (7.5)	ITT risk difference = 1.1% (-3.5, 1.3)	1 (0.1%)
	Control	1048	87 (8.6)	1012	87 (8.6)		4 (0.38%)
Patel 2015 (RCT) [14]	Intervention	554	60 (10.8) (day 14)	551	60 (10.9) (day 14)	Hospital vs community; ITT HR = 1.61 (1.16, 2.24), PP HR = 1.32 (0.93, 1.88)	1 (0.18%)
	Control	564	102 (18.1) (day 14)	534	102 (19.1) (day 14)		1 (0.18%)
Soofi 2012 (cRCT) [13]	Intervention	2529	187 (7.4)	2341	187 (8)	ITT risk difference = 5.2% (-13.7%, 3.3%).	2 (0.09%)
	Control	2162	273 (12.6)	2069	273 (13)		0
Observational studies							
Addo-Yobo 2011 [18]	All	873	76 (8.7)	823	76 (9.2)	N/A	0
Morre 2019 [41]	All	117	5 (4.3)	102	5 (4.9)	N/A	0
Onono 2018 [42]	All	1906	40 (2.1) (day 4)	1799	40 (2.2) (day 4)	N/A	5 (0.26%)

HR – hazards ratio, ITT – intention to treat, PP – per protocol, CI – confidence interval

*In all cases “intervention” refers to home-based management and ‘control’ refers to management in hospital.

†Day 6 treatment failure except where noted.

**Table 4:** Outcomes of studies with comparison groups other than home vs hospital management, or with primary outcomes other than treatment failure in patients with chest-indrawing pneumonia

Paper	Primary Outcome	Group	Primary Outcome (%)	Comparison (95% CI)	Mortality by day 6
**Randomised (RCT) and cluster-randomised controlled trials (cRCT)**					
Ashraf 2019 (RCT) [36]	Treatment success	Day Clinic	Day clinic alone = 184/235 (78.3), Day clinic plus hospital referral when needed = 220/235 (93.6).	Treatment success in day clinic or hospital alone: RR = 0.79 (0.65, 0.97). Referred onwards due to lack of success, 15% day clinic vs 9% hospital: RR = 1.28 (1.02, 1.60). Successfully managed when including referrals RR = 0.89 (0.62, 1.26).	0 during treatment, 3 over 6 mo follow-up.
		Hospital	Local hospital alone = 201/235 (85.5), Local hospital plus referral to a higher facility when needed = 223/235 (94.9)		2 (0.85%) during treatment, 4 over 6 mo follow-up.
Keitel 2019 (RCT) [39]	Treatment failure by day 7, or hospitalisation	ePOCT (including home management).	13/401 (3.2%) (of whom 7/401 had chest indrawing).	Treatment failure: risk difference = 1.9% (-3.7, -0.1%), RR = 0.60 (0.37, 0.98), hospitalisation: risk difference = -0.9 (-1.8, 0), RR = 0.33 (0.11, 1.02).	0
		ALMANACH (all patients go to hospital).	21/297 (7.1%)** (of whom 8/297 had chest indrawing).		2 (0.7%)
Tesfaye 2020 (cRCT) [43]	Severe pneumonia diagnosed	IMCI with pulse oximeter.	148/928 (15.9%), (95% CI = 4.7%, 27.2%)	Crude OR = 4.7 (1.9, 11.8)., aOR = 5.4, (2.0, 14.3).	2 (0.2%)
		IMCI without pulse oximeter.	34/876 (3.9%), (95% CI = 1.2%, 6.6%)		2 (0.2%)
**Observational studies**					
Ashraf 2008 [37]	Treatment success	Day Clinic	234/251 (93%)	N/A	0 during treatment, 4 over 3 mo follow up.
Chowdhury 2008 [16]	Appropriately managed	Intervention	1145/1271 (90%)	Crude OR = 16.1 (11.8, 22.1), OR adjusted for maternal age and household wealth = 15.7 (11.3, 21.8).	7 (0.6%)
		Historical control	94 /261 (36%)		3 (1.1%)
McCollum 2016 [40]	Was case referred if clinically eligible for referral?	All	Providers more than twice as likely to have referred a case who was clinically eligible for referral when the child had severe hypoxaemia than when they did not (84.3% (385/457) vs 41.5% (871/2099); *P* < 0.001.	N/A	Not recorded.

RR – relative risk, OR – odds ratio, aOR – adjusted odds ratio, mo – months

One other randomised trial from urban Bangladesh [36] found no difference in treatment outcomes of patients with chest-indrawing pneumonia and severe malnutrition treated in a day clinic compared to treatment in a local hospital, as long as patients were referred appropriately when needed. Subgroup analysis of a cluster-randomised trial in urban Tanzania [39] found that management of pneumonia with an algorithm which included oximetry measurements and point of care testing of CRP enabled more patients to be managed without antibiotics and in the community, with an improvement in outcomes. However, the number of patients with chest indrawing in each arm was low.

These trials reported low treatment failure rates for those treated in the community (median = 7.9%, range = 3.2-11.7), and mortality was very low (median = 0.9%, range = 0-0.2).

These trials included several important safety measures in selecting patients for community-based treatment and monitoring them and responding to deterioration (Table S2 in the **Online Supplementary Document**). Except for one focussing on children with malnutrition [36], trials generally excluded children with malnutrition or other chronic illnesses or comorbidities (including anaemia, wheeze, asthma), those who had already received treatment or were re-presenting, those who may be unable to comply with oral treatment (due to vomiting, for example), and anyone who would otherwise meet WHO criteria for severe pneumonia. Except for the two lady health worker programs in Pakistan, all trials included routine assessment with pulse oximetry to identify those with hypoxaemia (low blood oxygen level). After the initial visit, all studies required regular patient review for at least one week, ranging from daily to every 3-4 days. Studies included training for health care workers, including on how to counsel caregivers, although few studies reported this in detail.

### Other evidence on feasibility and appropriateness of community-based treatment

Observational studies from a range of African and Asia-Pacific contexts showed similarly low treatment failure rates (median = 7.0%, range = 2.2, 10) and very low mortality (range = 0%-0.6%) among children with chest-indrawing pneumonia treated in the community (**Table 3** and **Table 4**).

A qualitative synthesis of additional data on context, intervention, and processes identified additional lessons on the feasibility and appropriateness of community-based treatment of chest-indrawing pneumonia (Table S5 in the **Online Supplementary Document**).

The inclusion of pulse oximetry was found to improve pneumonia diagnosis and identification of hypoxaemia in a cluster randomised trial among rural primary care facilities in Ethiopia [43], an observational study among rural primary care facilities in Malawi [40], a secondary analysis of an RCT in urban Tanzania [39], and an observational study in urban Papua New Guinea [41]. The Malawi study found that, if health care workers had followed the revised WHO guidelines (in the absence of pulse oximetry), 42% of children with hypoxaemic pneumonia (SpO_2_<90%) would not have been referred compared to 8% using the existing guideline (which recommended referral for all those with chest indrawing) [40]. The Tanzania study found that pulse oximetry, alongside point-of-care CRP testing, better identified low-risk patients for outpatient treatment and was associated with better outcomes compared to usual IMCI care [39]. The Ethiopian study found increased adherence to treatment recommendations, including referral follow-through, but no significant effect on outcomes [43]. The Papua New Guinea study also found that pulse oximetry helped caregivers understand and trust care plans provided by health care workers [41].

While community health workers’ assessments and that of more highly trained health professionals were generally concordant [17,42], children with comorbidities, moderate malnutrition, or delays in care-seeking may be less likely to receive appropriate diagnosis and categorisation [42]. All studies included specific training on risk assessment for participating health care workers.

Studies found mixed effects of community-based treatment guidelines on the number and proportion of patients presenting, being referred, and adhering to treatment recommendations. An observational study in Bangladesh that introduced community-based management of chest-indrawing pneumonia after observing low referral completion rates found increased numbers of children presenting with chest-indrawing pneumonia to primary care facilities, lower referral initiation rates, and minimal change in the number or proportion following through with referral recommendations [16]. Other studies found low rates of referral completion (30%-47%), particularly among those in the hospital-care arm [13,43], and moderate rates (5%) of participants discharged from hospitals against medical advice [14].

The economic cost of management of chest indrawing pneumonia in the community, where examined, was significantly lower than the cost of inpatient management [14,36].

## DISCUSSION

Evidence from four trials suggests similar or lower treatment failure rates among children with chest-indrawing pneumonia treated at home compared to referral or inpatient management. However, these trials were all conducted in Pakistan or India and excluded patients with significant comorbidities, making generalisability limited – particularly in populations where malnutrition or other comorbidities are common and settings where the aetiology and associated features of pneumonia differ.

Evidence from other interventional and observational studies supports the feasibility of home management of chest-indrawing pneumonia, if safeguards are in place, with relatively low rates of treatment failure and mortality. These studies included settings with higher HIV prevalence and likely higher rates of bacterial pneumonia than peri-urban Pakistan and India, where the RCTs were conducted. They also represented care models based in the community, primary care facilities, secondary and tertiary hospitals, and “day clinics”. In all these studies, the conditions for safe outpatient management of chest-indrawing pneumonia were carefully defined. Children with hypoxaemia or danger signs and those with HIV, malnutrition, anaemia, or other comorbidities, were all managed as inpatients. A strong emphasis was placed on appropriate training of the health care staff, education of caregivers, and appropriate and timely follow-up of all patients.

Pulse oximetry was a core part of risk assessment in most included studies (although two of the earlier RCTs did not include oximetry), identifying severely ill children with hypoxaemia who otherwise may not have been appropriately referred or treated with oxygen. This finding concurs with other studies on pulse oximetry that have found pulse oximetry is an objective measure that improves hypoxaemia detection and risk stratification and provides health care workers and patients/families with greater confidence in treatment plans [40,44-49].

Aside from pulse oximetry, there is insufficient evidence supporting the inclusion of other point-of-care tests (eg, CRP) as part of routine triage of children with chest-indrawing pneumonia [39,50].

These findings are supported by a recent study on the management of chest-indrawing pneumonia by community health workers in Bangladesh, India, Ethiopia, and Malawi, which found equivalent treatment outcomes compared to facility-based care [51]. This study involved modification of community health worker guidelines (iCCM) to include pulse oximetry and allow community-based treatment for children with chest-indrawing pneumonia, using strict risk assessment and follow-up procedures and providing close supportive supervision (including 3-monthly refresher training).

### Implications and interpretation

Based on existing evidence, we suggest that home treatment of chest-indrawing pneumonia may be appropriate for low-risk patients with adequate safety and care structures (**Table 5**).

**Table 5:** Recommendations for home treatment of chest-indrawing pneumonia for children aged 2-59 mo

Home treatment of chest-indrawing pneumonia should only be recommended in children who are low risk and have adequate care and safety provisions.
Assessment of risk requires assessment of clinical severity, including the presence of danger signs and hypoxaemia, and the presence of comorbidities such as HIV, malnutrition, or anaemia. Severe pallor/anaemia or severe malnutrition should indicate that home treatment is not safe. Moderate pallor, undernutrition, or other comorbidities should raise caution.
Oximetry should be used to exclude hypoxaemia that may not be detected by clinical signs alone before home care is considered safe in children with pneumonia and chest indrawing. In general, SpO_2_<90% should indicate need for hospital admission, while SpO_2_ 90%-93% should raise caution.
Other factors to consider include caregiver understanding of treatment, signs of deterioration, and when to return for review; and caregiver ability to return for urgent or routine review, taking into account geographical distance, and the availability and affordability of transport.
Staff delivering care in the community must be adequately trained, equipped, and supported to provide this level of care, must be able to recognise indications for referral to secondary or tertiary care, and must be able to enact such referrals.
Clinical checklists could be used to support decision making about safety of care in the community (example checklist in Appendix S2 in the **Online Supplementary Document**).

Where it is safe and feasible, the management of chest-indrawing pneumonia at home has benefits. Home management is less costly to both the health services and to families, and usually more convenient and acceptable to families [52]. Home management options are particularly valuable in remote geographies and in populations who face substantial barriers in accessing hospital care, with opportunities to improve treatment adherence, including compliance with referrals when they are needed.

However, current community and primary care structures and processes in many LMIC contexts are unlikely to provide the required risk assessment and safety net without substantial investment. Clinical risk assessment is poorly taught and practised, with respiratory signs being one of the most consistently missed elements of existing IMCI practice [53-55]. Pulse oximeters are rarely available or used in primary care settings [40,43,46,56-58] despite inclusion in WHO primary care guidelines and “priority medical device” lists [59,60].

Evidence from the included studies suggests that training, adequate equipment, supportive supervision, and structures for referral and review are essential to implementing home care. The inclusion of a risk assessment checklist may provide additional practical support to health care workers, particularly lower-level health care workers who would not usually be managing more severely ill patients without a referral (Appendix S2 in the **Online Supplementary Document**).

Implementation of home care for children with chest-indrawing pneumonia will vary between contexts, with this review identifying examples from urban hospitals, rural health facilities, and community settings. The day clinic model presented in three of the papers from Bangladesh is interesting, enabling a hybrid inpatient/outpatient service where patients attend a facility (which has oxygen, suction, and other services) from 8 am to 5 pm each day for parenteral antibiotics, and return to their home at night. However, more research is needed on the use of this model in other contexts.

### Limitations

This review was limited by the number and quality of studies included. We identified few randomised trials, from narrow geographical and epidemiological settings, and their trial conditions may not reflect what is possible in actual practice. To address these issues, we included interventional and observational studies that could provide more ‘real-life’ data on the conditions required to provide safe care in the community. Future studies exploring the implementation of home management of chest-indrawing pneumonia in more routine practice settings and in diverse contexts will give greater clarity and confidence about how to safely manage these children without admission.

## CONCLUSIONS

Home treatment of chest-indrawing pneumonia can produce treatment outcomes comparable to hospital-based care for carefully selected populations in certain contexts. However, home treatment should only be recommended in children who are low risk and in contexts where adequate care and safety provisions are available. Prospective operational research into home treatment of chest indrawing pneumonia in settings outside of South Asia, particularly Sub-Saharan Africa, would help with determining the generalisability of the findings of existing trials.

## Additional material


Online Supplementary Document
