# Supplementary material for: Which children with chest-indrawing pneumonia can be safely treated at home, and under what conditions is it safe to do so? A systematic review of evidence from low- and middle-income countries
Source: J Glob Health. 2022 Aug 31;12:10008. doi: 10.7189/jogh.12.10008 (PMC9428503; doi:10.7189/jogh.12.10008)
Supplement: Online Supplementary Document [file jogh-12-10008-s001.pdf]

Supplemental material

Paper: Which children with chest-indrawing pneumonia in can be safely treated at home, and under what conditions is it safe to do so? A systematic review

Contents

Appendix S1 – Additional Methods detail 1
Information sources, search strategy and data collection details 1
MEDLINE search strategy 4
Table S1. Assessed strength and risk of bias of included studies, according to Effective Public Health Practice Project (EPHPP) ratings 7
Table S2. Contextual details on management of patients in included studies........ 7
Table S3. Numbers of patients screened and excluded, numbers of protocol violations and loss to follow up for all reviewed studies 11
Table S4. Definitions used for ‘treatment failure’ in studies which included treatment failure as a primary outcome 15
Table S5. Key outcomes or practice points, other than primary outcome for included studies........ 16
Appendix S2 – Risk Assessment Checklist Example questions caregiver to ask to help decide if patient can be safely treated in the 19
community 19

Appendix S1 – Additional Methods detail

Information sources, search strategy and data collection details

Information sources

Literature search strategies were developed using medical subject headings (MeSH) and text words related to acute respiratory infection, and oral antibiotic or outpatient treatment. We searched MEDLINE (OVID interface), EMBASE (OVID interface) and PubMed for all relevant published papers.

We scanned the reference lists of included studies or relevant review papers identified through the search.

Where appropriate, authors were contacted to get more information on the contexts in which their studies took place.

Search strategy

Both qualitative and quantitative studies were sought, published from the year 2000 onwards. No study design, or language limits were imposed on the search. The specific search strategies were created by a Health Sciences Librarian with expertise in systematic review searching. The MEDLINE strategy was developed with input from the project team. The MEDLINE search strategy is documented below. After the MEDLINE strategy was finalized, it was adapted to the syntax and subject headings of the other databases.

Study Records

Data management

All search results were saved in a shared file accessible to all members of the study team, and citations saved in EndNote. When reviewing papers for inclusion and exclusion records accessible to all team members were kept in the shared folder and on password protected computers.

Selection process

The titles and abstracts of all papers identified though the search were reviewed by two independent reviewers (CW, PW) for inclusion. We obtained full reports for all titles that appeared to meet the inclusion criteria or where there was any uncertainty.

Two independent reviewers (CW, PW) then screened the full text reports and decided whether these met the inclusion criteria. We sought additional information from study authors where necessary to resolve questions about eligibility. We resolved disagreement through discussion with the team. We recorded the reasons for excluding trials. Neither of the review authors were blind to the journal titles or to the study authors or institutions.

Data collection process

We had a standardised form of specific items (see below) with which 2 independent reviewers extracted data from each eligible study and entered into an excel spreadsheet (Microsoft, Redmond, US). Reviewers resolved disagreements by discussion, and unresolved disagreements were discussed with the whole team. We contacted study authors to resolve any uncertainties.

Data Items

For all papers included in the study we sought the following information regarding potential variables and context in which the study took place. We documented whether this data was available in the published papers or whether it was acquired from contact with the authors or other means. If we were unable to find this data this was also noted.

Study details

-Type of study
- Year of publication
- Year of study (date range)
- Geographic location(s) – WHO region, country, sub-national region
- Urban, rural or mixed
- Tertiary, provincial/state, or district hospital
- Purpose of study

- Intervention
- Comparison
- Inclusion criteria
- Exclusion criteria
- Details of training provided to health providers in study

Population
- Participant number
- Age (2-11 months, 1-4 years), median age - Sex
- Comorbidities
- malnutrition
- anaemia
- HIV
- asthma
- congenital heart disease
- other (specify where possible)
- Vaccination status of patient population

Health service aspects
- Has a doctor reviewed all patients enrolled?
- If so, is the doctor a paediatric specialist?
- Who would usually review patients in this setting typically?
- What antibiotics, if any, were given?
- What duration of antibiotics was given?

Monitoring and supportive care
- Pulse oximetry checked on all patients?
- Respiratory rate checked on all patients?
- Patients observed for how long on first presentation?
- Clearly defined indications for when patient needs admission?
- Clearly defined indications for escalation of care if needed?
- If patient being managed at home how frequent is outpatient review?
- If patient managed at home is there documented procedure for caregiver education?

Outcomes
For all papers included in the study we will seek the following information regarding outcomes.
The primary outcomes reviewed will be:
- Mortality rates
- Overall
- Age specific (2 months to 1 year, 1 to 5 years, over 5 years)
- If data available, subgroup analysis by o Presence/absence of wheeze o Presence of comorbidities o Aetiology if known
o Any other subgroup analysis performed? (specify) Secondary outcomes that we will review will be:
- Treatment failure rates o How was this defined in the study?
- Primary outcome of individual papers if different from above
- Secondary outcomes of individual papers if not covered by above

- Other issues raised regarding
- acceptability
- feasibility
- practice points
- required capacity

Risk of Bias

Included studies may or may not have a comparison group. To assess the quality of and risk of bias within included studies, the methodological quality of potential studies was assessed by two independent reviewers (CW, PW) using the Effective Public Healthcare Panacea Project (EPHPP) QA Tool. Using this tool, studies were rated as strong, moderate or weak with respect to selection bias, study design, confounders, blinding, data collection method, withdrawals and dropouts, and a global rating given. This was undertaken by two separate reviewers. Where there was disagreement, a third reviewer (HG) was used as an arbitrator. An assessment of publication bias was not undertaken for this review.

MEDLINE search strategy

Database: Ovid MEDLINE(R) ALL <1946 to August 24, 2020>

1. exp *Pneumonia/
2. ((respiratory adj3 (infection* or distress or failure or disease* or illness*)) or pneumonia or pneumonias or lung-inflammation* or lobitis or nonspecific-inflammatory-lung-disease* or peripneumonia or pleuropneumonia or pleuropneumonitis or pneumonic-lung* or pneumonic-pleurisy or pneumonic-pleuritis or pneumonitides or pneumonitis or pulmonal-inflammation* or pulmonaryinflammation* or pulmonic-inflammation* or bronchiolitis).tw,kf.
3. *Pneumococcal Infections/
4. exp *Bronchiolitis/
5. 1 or 2 or 3 or 4
6. (chest-wall-indrawing or chest-indrawing or sub-costal-indrawing or sub-costal-recession or subcostalindrawing or subcostal-recession or chest-recession or chest-wall-recession or severe).tw,kf.
7. (home or community or first-level or (primary adj3 care) or health-centre* or healthcenter*).tw,kf,hw.
8. *Ambulatory Care/ or *Outpatients/ or *comprehensive health care/ or *primary health care/
9. (outpatient* or ambulatory).tw,kf.
10. 7 or 8 or 9
11. *treatment outcome/ or exp *treatment failure/
12. exp *mortality/
13. *Death/ or *infant death/
14. mo.fs.
15. (mortalit* or death* or surviv* or fatal* or failure or failed).tw,kf.
16. exp *Recurrence/ or *survival rate/
17. 11 or 12 or 13 or 14 or 15 or 16

18. (infan* or toddler* or pre-schooler* or preschooler* or kinder or kinders or kindergarten* or kinderaged or boy or boys or girl or girls or child or children or childhood or pediatric* or paediatric* or adolescen* or youth or youths or teen or teens or teenage* or school-age* or schoolage* or schoolchild* or schoolgirl* or schoolboy*).af.

19. developing countries/

20. (austere or (limited adj2 resource*) or (low adj2 resource*) or (transitioning adj econom*) or (third adj world) or LMIC or LMICs or (lami adj countr*) or (transitional adj countr*) or (low adj gdp) or (low adj gnp) or (low adj gross adj domestic) or (low adj gross adj national) or ((emerging or developing or (low adj income) or (middle adj income) or (low adj3 middle) or underdeveloped or under-developed or (less* adj developed) or underserved or under-served or deprived or poor*) and (countr* or nation*1 or econom* or population or world))).tw,kf.

21. exp africa/

22. americas/ or exp caribbean region/ or exp central america/ or latin america/ or mexico/ or exp south america/

23. europe/ or exp europe, eastern/ or exp transcaucasia/

24. antarctic regions/ or exp atlantic islands/ or exp indian ocean islands/ or exp pacific islands/

25. New Guinea/

26. asia/ or exp asia, central/ or asia, southeastern/ or borneo/ or cambodia/ or east timor/ or indonesia/ or laos/ or malaysia/ or mekong valley/ or myanmar/ or philippines/ or thailand/ or vietnam/ or asia, western/ or bangladesh/ or bhutan/ or india/ or middle east/ or afghanistan/ or iran/ or iraq/ or jordan/ or lebanon/ or oman/ or saudi arabia/ or syria/ or turkey/ or yemen/ or nepal/ or pakistan/ or sri lanka/ or far east/ or china/ or tibet/ or exp korea/ or mongolia/

27. (Afghanistan or Albania or Algeria or Angola or Antigua or Argentina or Armenia* or Aruba or Azerbaijan or Bahrain or Bangladesh or Barbados or Barbuda or Belarus or Byelarus* or Byelorussian or Belorussian or Belorus* or Belize or Benin or Bhutan or Bolivia or Bosnia or Botswana or Brasil or Brazil or Bulgaria or (Burkina adj Fas*) or (Upper adj Volta) or Burma or Burundi or Cambodia or Khmer or Kampuchea or Cameron* or Cameroon* or (Cape adj Verde) or (Cabo adj Verde) or (Central adj African adj Republic) or Chad or Chile or China or Colombia or Comoros or (Comoro adj Island*) or Comores or Mayotte or Congo or Kongo or (Cook adj Island*) or (Costa adj Rica) or (Cote adj D'ivoire) or Croatia or Cuba or Cyprus or Czech* or Djibouti or Dominica or Dominican or (East adj Timor) or (East adj Timur) or Ecuador or Egypt or El-Salvador or (Equatorial adj Guinea) or Eritrea or Estonia or Ethiopia or Fiji or (French adj Somaliland) or Futuna or Gabon or (Gabonese adj Republic) or Gambia or Gaza or (Georgia* adj Republic) or Ghana or Grenada or Guam or Guatemala or Guinea or Guiana or Guyana or Haiti or Herzeg* or Hercegovina or Honduras or Hungary or India or Indonesia or Iran or Iraq or (Ivory adj Coast) or Jamaica or Jordan or Kazakh* or Kenya or Kiribati or Korea or Kosovo or (Kyrgyz adj Republic) or Kyrgyzstan or Kirghizia or Kirghiz or Kirgizstan or Laos or (Lao* adj2 Democratic adj Republic) or (Lao* adj PDR) or Latvia or Lebanon or Lesotho or Basutoland or Liberia or Libya or Lithuania or Macedonia or Madagascar or (Magalasy adj Republic) or Malawi or Malay* or Sabah or Sarawak or Maldives or Mali or (Marshall adj Island*) or Mauritania or Mauritius or (Agalega adj Island*) or Mexico or Micronesia or Moldov* or Mongolia or Montserrat or Montenegro or Morocco or Ifni or Mozambique or Myanma* or Namibia or Nauru or Nepal or (Netherlands adj Antilles) or (Dutch adj Antilles) or (New adj Guinea) or (New adj Caledonia) or Nicaragua or Niue or Niger or Nigeria or (Northern adj Mariana adj Island*) or Nyasaland or Oman or Pakistan or Palau or Panama or (Papua adj New adj Guinea) or PNG or Palestine or Paraguay or Peru or Philipines or Philippines or Phillipines or Phillippines or Poland or (Puerto adj Rico) or Yemen or Romania or Roumania or Rumania or Russia* or Rwanda or Ruanda or (Saint adj Kitts) or (St adj Kitts) or Nevis or (Saint adj Vincent) or (St adj Vincent) or Grenadines or Samoa* or (Navigator

adj Island*) or (Saint adj Lucia) or (St adj Lucia) or (Saint adj Helena) or (St adj Helena) or (Sao adj Tome) or (Saudi adj Arabia) or Senegal or Serbia or Seychelles or (Sierra adj Leone) or Slovenia or Slovak* or (South adj Africa) or (Solomon adj Island*) or Somalia or (Sri adj Lanka) or Ceylon or Sudan or Surinam* or Swaziland or Syria or Tajikistan or Tadzhikistan or Tadjikistan or Tadzhik or Tanzania or Thailand or Tibet or Timor-Leste or Togo or (Togolese adj Republic) or Tokelau or Tonga or Trinidad or Tobago or Tunisia or Turkey or Turkmenistan or Turkmen or Tuvalu or Uganda or Ukraine or Uruguay or Urundi or USSR or (Soviet adj Union) or "Union of Soviet Socialist Republics" or Uzbekistan or Vanuatu or (New adj Hebrides) or Venezuela or Vietnam or (Viet adj Nam) or (Wallis adj2 Futuna) or (United adj Arab adj Republic) or (West adj Bank) or (West adj Indies) or Yemen or Yugoslavia or Zaire or Zambia or Zimbabwe or Rhodesia).tw,kf.

28. (africa or americas or caribbean or (central adj America) or (latin adj America) or (south adj America) or (eastern adj Europe) or Transcaucasia or antarctic or (atlantic adj island*) or (indian adj ocean adj island*) or (pacific adj island*) or polynesia or (central adj asia) or (southeast* adj asia) or (south-east* adj asia) or borneo or mekong or (western adj asia) or (middle adj east) or (far adj east)).tw,kf.

29. 19 or 20 or 21 or 22 or 23 or 24 or 25 or 26 or 27 or 28

30. 5 and 6 and 10 and 17 and 18 and 29

Table S1. Assessed strength and risk of bias of included studies, according to Effective Public Health Practice Project (EPHPP) ratings

Paper	Selection Bias	Study design	Confounders	Blinding	Data collection methods	Withdrawal and drop-outs	Overall
Addo-Yobo 2011	moderate	weak	weak	weak	strong	strong	weak
Ashraf 2019	strong	strong	strong	moderate	moderate	strong	strong
Ashraf 2008	weak	weak	weak	weak	moderate	strong	weak
Bari 2011	strong	moderate	moderate	weak	moderate	strong	moderate
Chowdhury 2008	strong	moderate	moderate	weak	moderate	moderate	moderate
Hazir 2008	moderate	strong	moderate	weak	moderate	strong	moderate
Jahan 2018	weak	weak	weak	weak	weak	n/a	weak
Keitel 2019	strong	strong	strong	weak	moderate	strong	strong
McCollum 2016	strong	weak	moderate	weak	weak	Moderate	moderate
Morre 2019	strong	moderate	moderate	weak	moderate	strong	moderate
Onono 2018	strong	moderate	moderate	weak	moderate	strong	moderate
Patel 2015	weak	strong	moderate	weak	moderate	moderate	moderate
Soofi 2012	moderate	strong	moderate	weak	moderate	strong	moderate
Tesfaye 2020	strong	strong	moderate	weak	moderate	strong	strong

Table S2. Contextual details on management of patients in included studies

**Paper**	**Setting details**	**Vaccination status**	**Who sees patient in study setting**	**Oximetry checked? (threshold for referral / Rx)**	**Defined protocol with escalation procedures?**	**Patient observed for how long?**	**Frequency of follow up**	**Training of health providers**	**Education of caregivers**

Addo-Yobo 2011	4 Urban tertiary referral hospitals, 7 semi-rural primary care centres	96.1% up to date with vaccines	Initially seen by paediatrician or study physician, follow up by paediatricians, trained nursing staff or community workers	No	Yes	Screening, enrolment, assessment, and provision of medication took place within one hour	Days 1, 2, 3, 6 and 14	Community workers in Bangladesh received 1 week of IMCI training on pneumonia management and study procedures. Nurses in Ghana were trained in IMCI	Caregivers administered first dose of antibiotic under direct supervision. Caregivers educated in danger signs and advised to return if danger signs developed.
Ashraf 2019	Urban day clinic – well equipped, with 8 beds, has oxygen, suction, nebuliser, glucometer and weighing scales. 2km from referral hospital.		Clinic has one physician, two nurses and four healthcare workers	Yes – all patients (90%)	Yes	8 am till 5 pm daily	Daily for at least 5 days, then weekly for 2 weeks, then 2 weekly for 3 months, then monthly for 6 months.		Mothers were encouraged to breastfeed.
Ashraf 2008	Urban day clinic - 12 beds in two separate rooms, has oxygen, suction and a weighing scale		A doctor, two nurses, and four health workers (on site 08001700, on call beyond these hours).	Yes – all patients (90%)	Yes	8 am till 5 pm daily	Daily until clinically improved, then 2 weekly for 3 months	The health workers were trained to prepare and administer diets to the children	The health workers were trained to educate and motivate mothers to comply with treatments and follow-up
Bari 2011	Rural community health workers (lady health workers (LHWs)) A LHW works from her own home and attends to 150–200 families.		Lady health worker, clinically supervised by a lady health visitor. All treatment failures were verified on the same day by a study physician. Study physicians and study coordinators visted monthly and district, provincial, and federal point persons from the LHW programme visited quarterly.	No	Yes		Days 2, 3, 6, and 14	LHWs were trained to screen every child presenting to them with cough and difficulty breathing for enrolment.	
Chowdhury 2008	First-level facilities - The research team ensured that the equipment, supplies, and drugs needed to provide IMCI care were available in the intervention facilities at all times. No oxygen or IV antibiotics. No fees. 5–27 km by road from referral facilities.		Facilities usually staffed by two paramedics: one person with a 4 year basic clinical training and a female reproductive health worker with 18 months' training in maternal and child health and family-planning services. Visited monthly by trained IMCI supervisors (doctors).	No	Yes – though not strictly followed by all health workers		Not defined	All paramedics at facilities in the intervention group were trained in IMCI and were visited monthly by trained supervisors (who were physicians)	
Hazir 2008	Paediatric departments in urban tertiary-care facilities		Study physician	Not clear	Yes	Baseline assessment and provision of study medication took place within 1 h of randomisation	Days 1, 3, 6, and 14	Initial and in-service training and careful study oversight of study physicians	

Jahan 2018	Rural day clinic - Health & Family Welfare Centre (HFWC) – has beds, antibiotics, oxygen, nebulization, suction		Doctors and nurses at clinic	Yes	Not clear	All day	Not defined		
Keitel 2019	Outpatient departments of 3 urban district hospitals and 6 urban health centres. Area of relatively low malaria endemicity and low HIV prevalence	96% PCV<br><br>97% Hib	Study Clinician	Yes – in the ePOCT arm (90%)	Yes		Days 3 and 7, or at any time if the parent was concerned. Patients cured at day 3 were followed up by phone only on day 7.		
McCollum 2016	72 rural public-sector practitioners at 18 health centres and 38 community health workers (CHW), in areas with consistent health services, representative of paediatric health care in rural Malawi. None of the health centres provided oxygen.		Daily clinics run by nurses or non-physician clinicians (clinical officers or medical assistants). Salaried health surveillance assistants ran weekly village clinics	Yes – all pateints (92%)	Yes		Not defined	One day training based on videos and small-group practical sessions, run by a paediatric pulmonologist. Lessons on the use of oximeters, training to diagnose pneumonia according to the guidelines & with oximetry. Evaluation at conclusion of training and further training where necessary. All trainees retrained part way through study. Monthly supervisory visits by research team members.	
Morre 2019	Children´s outpatient department in urban teritiary hospital		Clinic staff	Yes – all patients (90%)	Yes	2–4 h	Day 2 and day 6		Video on recognizing danger signs, including severe respiratory distress, head nodding, cyanosis, poor feeding and convulsions. Assessment of understanding, and ability and willingness to return for follow-up. Taught how to give oral amoxicillin (how much, how often and cleanliness). Not sent home until understood how to give home treatment, what to monitor for and when to return

Onono 2018	Rural community health workers in 241 community health units in area with high levels of poverty, high HIV prevalence, and high under-5 mortality. Poor road and other transport access.		Community health worker. Supervision and evaluation from nurses in the study area. Free 24/7 decision support hotline set up to give assistance to the CHWs treating pneumonia, linking the pneumonia case to a trained nurse for physical case confirmation. The nurse traced the child within 24 hours and independently assessed and classified the child.	No	Yes		CHW follow up Day 3 and day 6. Study nurse follow up days 4 and 14.	CHWs previously trained over six weeks, with six days of iCCM training under CHW trainers of trainers experienced in iCCM and IMCI. Training included lectures, presentations, discussions, case studies, clinical demonstrations, practice, role plays, video shows and facility-based clinical attachments. Pre- and post-training assessments were conducted.<br><br>Diploma holding nurses in the study area underwent training in the WHO adapted Kenya IMCI curriculum as well as the iCCM curriculum facilitated by national, county and sub-county IMCI trainers	The CHW educated the caregiver on danger signs and advised the caregiver to take the child to the nearest health facility if any danger sign became apparent or if the child was unable to take the oral antibiotic
Patel 2015	6 urban referral hospitals	91.2% up to date with vaccines	Research physician	Yes – all patients	Yes		day 2, 3, 5, 8, and 14	Rigorous training and retraining of the research physicians using standard operating procedures was used to minimize the biases that may arise due to lack of uniformity in assessing clinical signs between treatment groups and across sites.	

Soofi 2012	Rural community health workers (lady health workers (LHWs)). A LHW works from her own home and attends to 150–200 families. Receive replenishment of commodities from basic health units or rural health centres on a monthly basis, undergo monthly refresher sessions. LHWs in both groups were provided with respiratory rate timers (stopwatches) and thermometers.		Lady health worker.<br><br>Validation of the assessment of pneumonia diagnosis by LHWs was done as per protocol within 48h by trained community health workers in all cases, and in a subset by study physicians. For 21% of participating children a physician visited the child within 48 h of LHW	No	Yes		Days 2, 3, 6, and 14	LHWs: Women with at least 8 years of formal schooling, are trained for 15 months to deliver care in community settings.<br><br>Additional enhanced acute respiratory infections training module and video to emphasise classification of acute respiratory infections according to the WHO algorithm, identification of danger signs, and home pneumonia case management with oral amoxicillin, led by faculty members of the university department of paediatrics with trainers of the LHW programme. Training sessions were organised for LHWs, data collectors, and study physicians. The LHWs in control clusters received a refresher training course that consisted of existing acute respiratory infection modules of national LHW programme curriculum.	Carers were urged to return to the LHW if signs of very severe pneumonia became apparent.
Tesfaye 2020	24 rural primary health centres	74% fully vaccinated, 26% partially vaccinated	Community health workers	Yes – in intervention arm only	Yes (92%)		Day 2, day 5 and day 14	Training on IMCI algorithm and how to use pulse oximetry, by an IMCItrained paediatrician, supported by a video-based exercise and practical session.	

Notes: CHW = community health worker; IMCI = integrated management of childhood illness; Hib = haemophilus influenzae vaccine; LHW = lady health worker; PCV = pneumococcal conjugate vaccine.

Table S3. Numbers of patients screened and excluded, numbers of protocol violations and loss to follow up for all reviewed studies

Paper	Group	Number screened	Number of patients excluded (% of total patients screened) and reasons for exclusions*	Number included for intention to treat analysis (% of total patients screened)	Loss to follow up	Protocol Violations	Number included for per protocol analysis (% of total patients screened)

Addo-Yobo 2011	All	6583	Total 5709 (86.7)<br><br>Medical reasons:<br>1653 3 or more episodes of wheezing<br>1229 LCI resolves after bronchodilator<br>1090 Very severe disease<br>904 Known prior episodes of asthma<br>545 Hospitalization in the last 2 weeks<br>274 Other diseases requiring antibiotics<br>226 Known chronic condition<br>163 Severe malnutrition<br>114 Persistent vomiting<br>15 Anaphylactic reaction<br>10 Severe pneumonia with measles<br>9 Kerosene ingestion<br>4 Near drowning<br><br>Non-medical reasons:<br>2230 Living outside study area<br>395 Refusal to participate in study<br>45 Previous inclusion in the study or other study<br>20 Judgment that the child won't be able to complete study	873 (13.3)	22	28	823 (12.5)
Ashraf 2019	Day clinic	1338	Total 868 (64.9)<br><br>653 "did not meet inclusion criteria"<br>144 no consent<br>71 "other reasons"	235 (17.6)	0	15	220 (16.4)
	Hospital			235 (17.6)	0	10	225 (16.8)
Ashraf 2008	All	557	Total 306 (54.9)<br><br>92 non-severe pneumonia<br>57 very severe pneumonia (of which 48 severely malnourished) 80 bronchiolitis<br>14 contagious diseases eg measles/TB<br>13 had received antibiotics<br>13 no consent<br>9 congenital heart disease<br>9 lived outside area<br>8 presented too late in day<br>10 other reasons	251 (45.1)	0	6	245 (44.0)

Bari 2011	Intervention	11230	Total 9235 (82.2)<br><br>9201 non-severe pneumonia<br>9 very severe pneumonia<br>1 severe malnutrition<br>4 carers declined<br>20 already taking antibiotic(s)	1995 (10.3)	47	91	1857 (9.6)
	Control	8061	Total 6584 (81.7)<br><br>6548 non-severe pneumonia<br>6 very severe pneumonia<br>2 severe malnutrition<br>12 carers declined<br>16 already taking antibiotics	1477 (7.7))	36	87	1354 (7.0)
Chowdhury 2008	Intervention	22722	Total 21451 (94.4)<br><br>21045 non-severe pneumonia 33 other severe classifications<br>373 not able to contact	1455 (4.2)	n/a	n/a	1455 (4.2)
	Historical Control	11552	11291 (97.7)<br><br>11180 non-severe pneumonia 20 other severe classifications<br>91 not able to contact	261 (0.8)	n/a	n/a	1455 (4.2)
Hazir 2008	Intervention	6901	Total 4801 (69.6)<br><br>1273 known asthmatic<br>874 very severe pneumonia/disease<br>836 living outside area<br>422 chest indrawing resolved with bronchodilators<br>415 comorbid conditions<br>349 refused to participate<br>258 previous inclusion in the study<br>215 severe malnutrition<br>191 hospitalisation in the last 2 weeks<br>68 persistent vomiting<br>17 known anaphylaxis to antibiotic	1052 (15.2)	6	21	1025 (14.9)
	Control			1048 (15.2)	13	23	1012 (14.7)

Keitel 2019	ePOCT protocol (treat at home)	4729	Total 1537 (32.5)<br><br>1391 not eligible<br>146 eligible but not recruited<br>1456 severe symptoms or no cough<br>923 without tachypnoea or chest indrawing<br>115 malaria rapid test positive	401 (8.5)	3	5	393 (8.3)
	ALMANACH protocol (refer to hospital)			297 (6.3)	4	5	288 (6.1)
McCollum 2016	All	14092	Total 826 (5.9)<br><br>826 failed oxygen saturation measurement	13266 (94.1)	n/a	n/a	13266 (94.1)
Morre 2019	All	120	Total 3 (2.5)<br><br>2 severe pneumonia<br>1 moderate pneumonia with acute gastroenteritis	117 (97.5)	15	0	102 (85)
Onono 2018	All	11676	Total 9770 (83.7)<br><br>8520 pneumonia without chest indrawing<br>1250 referred for danger signs	1906 (16.3)	107	0	1799 (15.4)
Patel 2015	Intervention	6634	Total 5516 (83.1)<br><br>4060 no consent<br>1489 no lower chest indrawing<br>743 chest indrawing resolved with bronchodilators<br>462 not 3-59 months<br>431 antibiotics for 48 hours or more prior to admission<br>358 presence of danger sign<br>308 living outside area<br>267 history of >3 wheezing episodes or diagnosed asthma<br>224 chronic conditions<br>163 clinically severe malnutrition<br>127 hospitalisation >48 hours in last 2 weeks<br>45 radiological consolidation, effusion or pneumothorax 445 other (see paper)	554 (8.4)	3	0	551 (8.3)
	Control			564 (8.5)	30	0	534 (8.0)
Soofi 2012	Intervention	10690	Total 5999 (56.1)<br><br>5792 non-severe pneumonia<br>117 no lower chest indrawing<br>30 already enrolled<br>21 already receiving antibiotics<br>15 asthma, TB or diarrhoea<br>14 severe malnutrition<br>4 no consent<br>4 very severe disease	2529 (23.7)	159	29	2341 (21.9)
	Control			2162 (20.2)	89	4	2069 (19.4)

			2 too old				
Tesfaye 2020	IMCI with pulse oximeter	2600	Total 1672 (38.4)<br><br>1667 no cough<br>5 age less than 2 months	928 (21.3)	66	Not recorded	862 (19.8)
	IMCI without pulse oximeter	1755<br><br>4355	Total 879 (20.2)<br><br>817 no cough<br>38 age less than 2 months<br>18 Cough >14 days	876 (20.1)	47	Not recorded	829 (19.0)

*total numbers of reasons for exclusion do not always add up to total number of patients excluded as some patients had multiple reasons for exclusion

Table S4. Definitions used for 'treatment failure' in studies which included treatment failure as a primary outcome

**Study**	**Definition of treatment failure**
Addo-Yobo 2011	Any of the following: clinical deterioration occurring any time after enrolment, inability to take oral medication due to persisting vomiting as assessed by study physician, change or addition of antibiotics - indications for this were 1) Developing a co-morbid condition, 2) Persistence of fever > 38°C with lower chest indrawing on day 3 (after 72 hours), 3) Either fever or lower chest indrawing alone at day 6 or later, serious adverse event considered possibly or probably related to amoxicillin
Bari 2011	Defined as the appearance of a danger sign (unable to drink or breastfeed, convulsions, vomiting after ingestion of food or drink, and abnormally sleepy or difficult to wake), temperature at least 100°F and lower chest indrawing on day 3, fever or lower chest indrawing alone on day 6, and change of antibiotic (through self-referral or by carers).
Hazir 2008	Any of the following: clinical deterioration (Development of danger signs such as inability to drink, abnormal sleepiness, central cyanosis, and convulsions), inability to take oral medication due to persistent vomiting (defined as vomiting three repeated doses of oral amoxicillin within 0·5 h of administration), development of a comorbid condition requiring an antibiotic (defined as development of a disease requiring antibiotic treatment (eg, meningitis, dysentery, osteomyelitis, septic arthritis, evident tuberculosis), persistence of fever >38°C with lower chest indrawing (LCI) from day 3 to day 6; either fever or lower chest indrawing alone at day 6; hospitalisation related to pneumonia; serious adverse event; left against medical advice or lost to follow-up; voluntary withdrawal of consent; death.
Keitel 2019	At Any Time Between Initial Assessment and Day 7: Severe disease: Coma, >2 convulsions within 24 h, Inability to drink or breastfeed, Hypoxemia (SaO2 <90%), Severe tachypnea (Respiratory rate ≥97th percentile for age and temperature) , Severe tachycardia (Heart rate ≥90th percentile for age and temperature). At Day 3: Clinical pneumonia: Cough and tachypnea (espiratory rate ≥60 breaths/minute and age <12 months or respiratory rate ≥50 breaths/minute and age ≥12 months.), Cough and lower chest indrawing, Significant dehydration (Dehydration requiring facility-based treatment.). At Day 7: Fever or temperature ≥38, Clinical pneumonia: Cough and tachypnea (Respiratory rate ≥60 breaths/minute and age <12 months or respiratory rate ≥50 breaths/minute and age ≥12 months. Cough and lower chest indrawing. Diarrhoea (Three or more liquid stools per day.), Significant dehydration (Dehydration requiring facility-based treatment.), Serious skin infection (Skin infection requiring systemic antibiotic treatment and/or facility-based treatment.). A new significant symptom or sign related to the acute episode but not present at day 0.

Morre 2019	Deterioration in the child's condition necessitating admission to hospital, if signs of pneumonia with chest indrawing persisted on Day 6 or if the child died at any time during the study or in the month from enrolment.
Onono 2018	a) Appearance of any signs of pneumonia with danger signs such as inability to breastfeed or drink, lethargy or reduced level of consciousness, convulsions b) Change of antibiotic reatment or use of additional antibiotics beyond those prescribed and dispensed by the CHW c) Persistence of fever ≥38°C with lower chest indrawing on day 4 d) Presence of fever ≥38°C or lower chest indrawing or fast breathing at day 14 (relapse) e) Death
Patel 2015	Presence of any one of the following conditions - clinical deterioration of disease any time after enrolment that required change of antibiotics, hospitalization (any time for the children in the home managed group or clinical decision to extend the hospitalization longer than 48 h in the hospitalized group), an occurrence of a serious adverse event related to amoxicillin, left against medical advice (LAMA), voluntary withdrawal of consent from the study, or loss to follow up. Clinical deterioration was defined as appearance of signs of very severe disease such as persistent vomiting (vomiting repeated three times within an hour due to any reason), central cyanosis, grunt, stridor, abnormal sleepiness or difficulty to wake, inability to drink, SpO2 < 85 %, convulsions, or death. Antibiotics would be changed if there was clinical deterioration, developing a co-morbid condition, or, persisting fever > 98.6 °F with lower chest indrawing even after 3rd day, or, fever alone at day 5, or, lower chest indrawing alone (non responsive to three doses of nebulisation with bronchodilator) at day 5 (as reported by the mother), or, persistence of fast breathing after day 7 which is unresponsive to three doses of nebulization with bronchodilator.
Soofi 2012	Any of the following: Appearance of any signs of very severe pneumonia; Change of antibiotic treatment without objective criteria of treatment failure; Persistence of fever greater than 38°C with lower chest indrawing on day 3 (after 48 h of initiation of treatment); Either fever greater than 38°C or lower chest indrawing alone at day 6; Death

Table S5. Key outcomes or practice points, other than primary outcome for included studies

Paper	Key outcomes or practice points, other than primary outcome
Addo-Yobo 2011	• Relapse rate day 14 among those cured on day 6 20/733 (2.7%, 95% CI 1.5 - 3.9)<br>• Treatment failure rate ranged from 6.4% in Ghana to 13.2% in Vietnam<br>• Persistence of chest indrawing on day 6 most common reason for failure, which was most common in Bangladesh
Ashraf 2019	• Referred-readmitted during 6-months' follow-up period 13% vs 15% RR 0.89 (0.67–1.18) P 0.21<br>• The mean (± SD) time (days) required to treatment success with day clinic was marginally longer compared to hospital care [7.9 (5.5) vs. 7.1 (3.1), P = 0.04]<br>• The average societal cost per child treated was 34.4% lower for day clinic (US$ 184.27 ± 11.7) than for hospital care (US$ 280.88 ± 13.6). Including all clinical procedures as well as follow-up, the average provider cost per child was US$165.17 ± 30.29 for DCA and US$252.08 ± 24.20 in hospital
Ashraf 2008	• Reasons for referral to hospital were respiratory distress with hypoxaemia in ten patients and heart failure secondary to ventricular septal defect (VSD) in one patient
Bari 2011	• 54 (2%) of 2677 children who were well on day 6 relapsed between days 6 and 14, with a non-significant difference in proportions between each group<br>• High concordance in diagnosis of severe pneumonia between the LHW and an independent assessor (94%)<br>• In the intervention group compliance was more than 93% at all timepoints. 92% of children in the control group who were referred after an initial dose of co-trimoxazole complied with referral, but only 1% of those referred were admitted to hospital.<br>• "Community case management of severe pneumonia by LHWs using oral amoxicillin was well accepted by carers and enthusiastically adopted by the LHWs"

Chowdhury 2008	• The median duration of illness after the visit to the first-level facility was 6 days (IQR 4–8) in the fi rst cohort and 5 days (4–7) in the second.<br>• After introduction of the modified guidelines, the proportion of severe pneumonia cases who were referred to higher level facilities was reduced from 245 (94%) of 261 children to 107 (8%) of 1271 children (p<0·0001)<br>• The rate of compliance with referral was 36% before modification of the guidelines, compared with 47% after modification (p=0·047)<br>• The proportion of children with danger signs or stridor who complied with referral increased from 41% before modification to 52% afterwards<br>• The number of children with severe pneumonia who presented to the first-level facilities was nearly five times greater after modification of the guidelines than before (372 to 1677). We did not notice such an increase in use of facilities in the comparison group of the parent MCE study. Modification of the guidelines, which allowed severe cases to be treated locally rather than referred, could have contributed to the increased use.
Hazir 2008	• 56 (3%) of 1873 children who were well on day 6 relapsed between days 6 and 14, with non-significant difference in proportions between each group<br>• Additional analyses in subgroups with diarrhoea, vomiting, audible wheezing, or previous antibiotic found little effect on the estimate of the effect of ambulatory versus hospitalised treatment.<br>• Young infancy (age 3–5 months compared with >12 months), underweight for age, and very fast breathing were predictive of treatment failure by day 6 in a multivariate logistic regression model.<br>• In children under the age of 24 months, being breastfed at presentation was protective. Antibiotic use in the 7 days before randomisation and auscultatory wheeze were predictive of treatment failure at day 6
Keitel 2019	• 0.2% (2/865) of patients in the ePOCT arm vs 1.4% (12/854) of patients in the ALMANACH arm experienced "severe disease" as per the clinical failure criteria at follow-up (RR, 0.16 [95% CI, .04–.73]
McCollum 2016	• Nearly 94% (1222) of the 1301 successful measurements made by providers were within two percentage points of the expert's measurements. The weighted kappa for the overall level of agreement between the providers and the expert (0.41; Table 2) indicated moderate agreement.<br>• 27% (53/195; P < 0.001 of children clinically ineligible for referral had severe hypoxaemia.<br>• Compared with the facility-based providers, the CHW correctly referred a greater proportion of severely hypoxaemic children who did not have indrawing or general danger signs (67.3% [35/52] vs 12.6% [18/143]; P < 0.001) and a lower proportion of children with either indrawing or danger signs and an oxygen saturation of at least 90% (14.0% [127/906] vs 62.3% [744/1193]; P < 0.001).
	• If the facility-based providers had followed the WHO 2014 guidelines, 390/928 (42.0%) children eligible for referral (390/568 [68.7%] of severely hypoxaemic children), or 861/1399 (61.5%) children eligible for referral (861/1111 [77.5%] moderately or severely hypoxaemic children), would not have been referred.<br>• If the same providers had followed the latest relevant Malawian guidelines, which do recommend referral because of chest indrawing alone, 143/1761 (8.1%) children eligible for referral (143/568 [25.2%] severely hypoxaemic children) – or 425/2043 (20.8%) children eligible for referral (425/1111 [38.3%] moderately or severely hypoxaemic children) – would not have been referred<br>• If the trained CHW had followed either the WHO 2014 or the Malawian 2000 guidelines, 52/990 (5.3%) children eligible for referral (52/84 [61.9%] severely hypoxaemic children) – or 419/1357 (30.9%) children eligible for referral (419/711 [58.9%] moderately or severely hypoxaemic children) – would not have been referred in the absence of oximetry
Morre 2019	• After watching the video, all parents were able to recall danger signs on Day 1 and Day 2<br>• On Day 2 and Day 6, 98 and 97 mothers, respectively, demonstrated that they could administer amoxicillin correctly.<br>• Two mothers on Day 2 had not given the antibiotic at all.<br>• Pulse oximetry was considered useful in detecting hypoxemia and screening children for suitability for outpatient treatment, and helped parents understand that their child was being carefully monitored.
Onono 2018	• Cumulative treatment failure by day 14 n=147 (7.7%)<br>• Comparison between CHWs and nurses for concordance in identification, classification and treatment of lower chest indrawing showed an agreement of 88.7% with a Kappa coefficient of 0.65 (95% CI: 0.57–0.62; exact p-value <0.001).

	• Children with moderate malnutrition (OR 1.68; 95% CI: 1.22–2.30), comorbidities such as diarrhoea or malaria (OR 1.55; 95% CI: 1.32–1.81) or those with an additional day of delay in presenting to the CHW (OR 1.06, 95% CI:1.02–1.10) were more likely to have an incorrect classification of lower chest indrawing by the CHW
Patel 2015	• Clinical deterioration at < 7 days was not significantly different between the groups<br>• The hospital group was significantly more likely than home children to fail treatment at any time point. (HR 1.79; 95 % C.I. 1.30, 2.46, p < 0.01).<br>• The per protocol analysis, though tended to show a similar trend (RR 1.32), was statistically non-significant (p = 0.10).<br>• Failures due to left against medical advice or voluntary withdrawal were significantly more in hospital than the home group. [5.3 % (30/564) vs 0.5 % (3/554); p < 0.001]<br>• The most common reason for exclusion in this study at screening was refusal of consent to participate if the child were to be randomized to hospital group<br>• The median total cost for treating at home was significantly less than treating at hospital for the first 48 h. (Rs 399 for home vs. Rs 602 for the hospital group, p < 0.001)
Soofi 2012	• 49 (1.25%) of 3930 children who were well on day 6 relapsed between days 6 and 14, with a non-significant difference in proportions between each group<br>• 30% of carers of children in the control group refused referral<br>• Concordance between LHW and physician diagnosis was 99·1% within 12 h, 96·5% between 13–24 h, and 61·5% between 25–48 h. LHWs had high specificity for detecting the presence of severe pneumonia.
Tesfaye 2020	• The proportion of treatment failure at day 14 was 132/928 (14.2%, 95%CI 6.0 to 22.4) in the intervention arm and 93/876 (10.6%, 95%CI 5.2 to 16.1) in the control arm (p=0.622).<br>• The COR of treatment failure for children with oxygen saturation <90% was 3.3 (95% CI 1.87 to 5.80) as compared with children with oxygen saturation greater or equal to 90%.<br>• After examining the clustering effect, the proportion of children with severe pneumonia referred to the hospital was 116/148 (78.4%, 95% CI 67.6 to 89.2) in the intervention arm and 15/34 (44.1%, 95% CI 6.9 to 81.3) in the control arm, with p=0.496<br>• Among these, 62/116 (53.4%) in the intervention arm and 11/15 (73.3%) in the control arm, reached the hospital and received the standard treatment<br>• The impact of pulse oximeter is not modified by the health professionals' medical background knowledge for diagnosing severe childhood pneumonia (p value=0.828).<br>• In subgroup analysis for the intervention arm of 135 children with hypoxaemia, 56 of them did not have chest in-drawing. Pulse oximetry identified 67% of children without chest indrawing or danger signs<br>• There was no difference in treatment failure between the trial arms. A large number of children from the intervention arm did not go to hospital. Limited access to transport was the main reason for low compliance with referral in the study settings. This implies that only providing pulse oximetry may be insufficient to improve treatment outcome

Appendix S2 – Risk Assessment Checklist Example questions caregiver to ask to help decide if patient can be safely treated in the community

1. Is the child less than 2 months of age?
2. Does the child have any other known or suspected illnesses, including HIV, heart disease, or anaemia?
3. Is the child's weight inappropriately low for their height or age, per WHO charts?
4. Does the child have any general or pneumonia danger signs as per IMCI?
5. Is the child's oxygen saturation less than 92%, or do they have cyanosis?
6. Does the child look pale, or otherwise unwell?
7. Does the child's caregiver have any difficulty understanding how to give the treatment or when to bring the child back for review?
8. Are there other reasons such as finances or transport availability that it would be difficult for the caregiver to urgently bring the child back for review if needed?
9. Do you have any other concerns that the child will not receive adequate treatment at home, or do you think for any other reason that the child is likely to get worse if treated at home?

IF the answer to any of the above questions is YES, it is likely this patient will be at higher risk if treated at home, and inpatient referral and management should be strongly considered.
